# Progression of severity in coronavirus disease 2019 patients before treatment and a self-assessment scale to predict disease severity

**DOI:** 10.1186/s12879-022-07386-3

**Published:** 2022-04-26

**Authors:** Ye Yao, Jie Tian, Xia Meng, Haidong Kan, Lian Zhou, Weibing Wang

**Affiliations:** 1 Department of Biostatics, School of Public Health, Fudan University, Shanghai, 200032 China; 2 School of Public Health & Shanghai Institute of Infectious Disease and Biosecurity, Fudan University, Shanghai, 200032 China; 3 Department of Environmental Health, School of Public Health, Fudan University, Shanghai, 200032 China; 4 Key Laboratory of Public Health Safety of Ministry of Education, Fudan University, Shanghai, 200032 China; 5 Jiangsu Provincial Center for Disease Control and Prevention, No. 172 Jiangsu Road, Gulou District, Nanjing, 210009 China

**Keywords:** Coronavirus Disease 2019, Self-Assessment Scale, Severity

## Abstract

**Objectives:**

This study aims to further investigate the association of COVID-19 disease severity with numerous patient characteristics, and to develop a convenient severity prediction scale for use in self-assessment at home or in preliminary screening in community healthcare settings.

**Setting and participants:**

Data from 45,450 patients infected with COVID-19 from January 1 to February 27, 2020 were extracted from the municipal Notifiable Disease Report System in Wuhan, China.

**Primary and secondary outcome measures:**

We categorized COVID-19 disease severity, based on The Chinese Diagnosis and Treatment Protocol for COVID-19, as “nonsevere” (which grouped asymptomatic, mild, and ordinary disease) versus “severe” (grouping severe and critical illness).

**Results:**

Twelve scale items—age, gender, illness duration, dyspnea, shortness of breath (clinical evidence of altered breathing), hypertension, pulmonary disease, diabetes, cardio/cerebrovascular disease, number of comorbidities, neutrophil percentage, and lymphocyte percentage—were identified and showed good predictive ability (area under the curve = 0·72). After excluding the community healthcare laboratory parameters, the remaining model (the final self-assessment scale) showed similar area under the curve (= 0·71).

**Conclusions:**

Our COVID-19 severity self-assessment scale can be used by patients in the community to predict their risk of developing severe illness and the need for further medical assistance. The tool is also practical for use in preliminary screening in community healthcare settings.

**Summary:**

Our study constructed a COVID-19 severity self-assessment scale that can be used by patients in the community to predict their risk of developing severe illness and the need for further medical assistance.

**Supplementary Information:**

The online version contains supplementary material available at 10.1186/s12879-022-07386-3.

## Strengths and limitations of this study

- Our COVID-19 severity self-assessment scale can be used by patients in the community to predict their risk of developing severe illness and the need for further medical assistance.
- The tool is also practical for use in preliminary screening in community healthcare settings.
- The data used in the scale construction were entirely from China, which could potentially limit the generalizability of the findings and use of the scale in other countries.

## Introduction

Coronavirus Disease 19 (COVID-19) has formed a worldwide pandemic [1], and its clinical spectrum of disease ranges from mild to critical illness. Most COVID-19 patients present with mild symptoms, such as fever and cough, but a small proportion of patients develop severe pneumonia with progression to life-threatening complications, including acute respiratory distress syndrome, multi-organ failure, and death [2]. Consequently, the case-fatality rate differs widely between patients with severe and nonsevere disease. According to the most comprehensive report from the Chinese Centre for Disease Control and Prevention, which reviewed 72,314 cases, the average COVID-19 case-fatality rate is 2.3%, but it is as high as 49% in patients with critical illness [3].

In some countries, such as the United States [4], the Republic of Korea [5], and Scotland, differing case-fatality rates and limited resources have led to the adoption of separate management strategies for severe and nonsevere disease. Particularly in regions with large number of cases, because of the need to conserve the number of available intensive care unit beds and ventilators, the government has dictated home quarantine for patients with nonsevere disease to reserve hospitalization for patients with severe disease [6–8]; however, the patient who inappropriately quarantined at home have a certain chance of sudden deterioration, which may lead to a very poor prognosis if they can’t see a doctor in time. For patients in quarantine in community, accurate COVID-19 severity self-assessment can guide appropriate and timely medical consultation. During this pandemic, biological and clinical predictors of COVID-19 infection severity will also assist in the judicious allocation of limited resources.

Previous research has shown that clinical characteristics, including chronic disease, lymphopenia, and elevated D-dimer level, are associated with the severity of COVID-19 [9–15], but most of these studies used univariate analysis with comparatively complicated laboratory parameters. A study among hospitalized adults identified through the United States COVID-19-Associated Hospitalization Surveillance Network (COVID-NET) showed that increasing age, male sex, and underlying conditions were associated with higher risk of ICU admission and death [16], but it failed to include mild patients who were not hospitalized. Wynants et al. [17] and Urwin et al. [18] presented information available at that time on prediction models on prognosis of COVID-19. Although those models reported good to excellent predictive performance, they were limited in small sample size or rated at high risk of bias. Further evidence needs to emerge around the validity of these scores. This study aims to further investigate the association of COVID-19 disease severity with numerous patient characteristics, and to develop a convenient severity prediction scale for use in self-assessment at home or in preliminary screening in community healthcare settings.

## Methods

### Data sources and processing

COVID-19 patient data for the period January 1 to February 27, 2020 were extracted from the municipal Notifiable Disease Report System, in Wuhan, Hubei Province. The data was obtained by investigations conducted by epidemiology professionals after the patient was diagnosed. The inclusion criterion was a confirmed COVID-19 diagnosis by positive high-throughput sequencing or reverse-transcription polymerase chain reaction assay of nasal and pharyngeal swab specimens. The study was conducted according to the principles of the Declaration of Helsinki, and all data were de-identified to protect patient confidentiality. Ethical approval for this study was obtained from the School of Public Health, Fudan University.

### Outcome measurement

The Chinese Diagnosis and Treatment Protocol for COVID-19 [19] defines four levels of COVID-19 disease: mild, ordinary, severe, and critical illness; additionally, asymptomatic infection is recognized. The specific definitions of each category are detailed in Additional file 1. We categorized COVID-19 disease severity, based on these definitions, as “nonsevere” (which grouped asymptomatic, mild, and ordinary disease) versus “severe” (grouping severe and critical illness).

### Potential predictive variables

The study variables included demographic factors (age, gender, participant location at enrollment); present/past medical history (illness duration, presence of hypertension, pulmonary disease, diabetes mellitus, cardio/cerebrovascular disease, chronic liver disease, and chronic kidney disease); clinical symptoms; blood test parameters (white blood cell count, lymphocyte count and percentage, and neutrophil percentage); and imaging findings (abnormal chest computed tomography scan).

According to the situation in Wuhan, the patient location at time of enrollment is divided into two categories: those staying at home, including those who have not received any medical services and are found at home; the hospitalized patients, including those who were hospitalized or confined to a location outside of their home (mobile cabin hospital or collection spot like a hotel). We collected the comorbid state by patient report and confirmed by the investigation of epidemiological staff. Specific definitions are detailed in Additional file 1.

### Statistical analysis

In all, 36 variables were considered as potential predictors of COVID-19 disease severity. T-test and chi-square test were used to compare the differences of each variable in patients with severe and nonsevere disease. The variables that showed significant association with disease severity were then included into logistic regression models for multivariable analysis, to confirm their candidacy for inclusion in the new prediction scale (COVID-19 Severity Self-Assessment Scale). In the logistic modelling, we used the following formulae to calculate the probability and 95% confidence intervals (CIs) [20].

- probability $$=exp\left(\Sigma \beta \times X\right)/\left [1+exp\left(\Sigma \beta \times X\right)\right]$$
- lower limit of 95%CI $=exp\left [\Sigma {X}_{n}\times {\beta }_{n}-\Sigma z\times SE\left(\beta \right)\right]/\{1+exp\left [\Sigma {X}_{n}\times {\beta }_{n}-\Sigma z\times SE\left(\beta \right)\right]$
- upper limit of 95%CI $=exp\left [\Sigma {X}_{n}\times {\beta }_{n}+\Sigma z\times SE\left(\beta \right)\right]/\{1+exp\left [\Sigma {X}_{n}\times {\beta }_{n}+\Sigma z\times SE\left(\beta \right)\right]\}$

Receiver-operator characteristic (ROC) analysis was performed and the area under the curve (AUC) calculated to verify the accuracy of the final prediction scale. We extracted p-values for AUC by conducting permutation analyses. To avoid the influence of potential collinearity among the variables, least absolute shrinkage and selection operator (LASSO) regression analysis was also performed.

The statistical analysis was performed using MATLAB software, version 2019b (MathWorks Inc). In this study, a p-value less than 0·01 is statistically significant.

### Patient and public involvement

No patient involved.

## Results

### Demographic characteristics

Data were collected from 45,450 patients. The study population had a mean (standard deviation [SD]) age of 53·44 (16·38) years, and 21,689 (47·7%) patients were men. The mean (SD) illness duration was 10·40 (7·90) days (see Additional file 1: Figure S1 data for the distribution). Among all the patients, 7,798 (17·2%) were considered to have severe disease and 37,652 (82·8%) to have nonsevere disease. Accordingly, 37,654 (82·9%) patients had already been under control (hospitalized or confined to a location outside of their home such as mobile cabin hospital and collection spot like hotel), and other were found at home because the lack of medical resource.

**Fig. 1:**
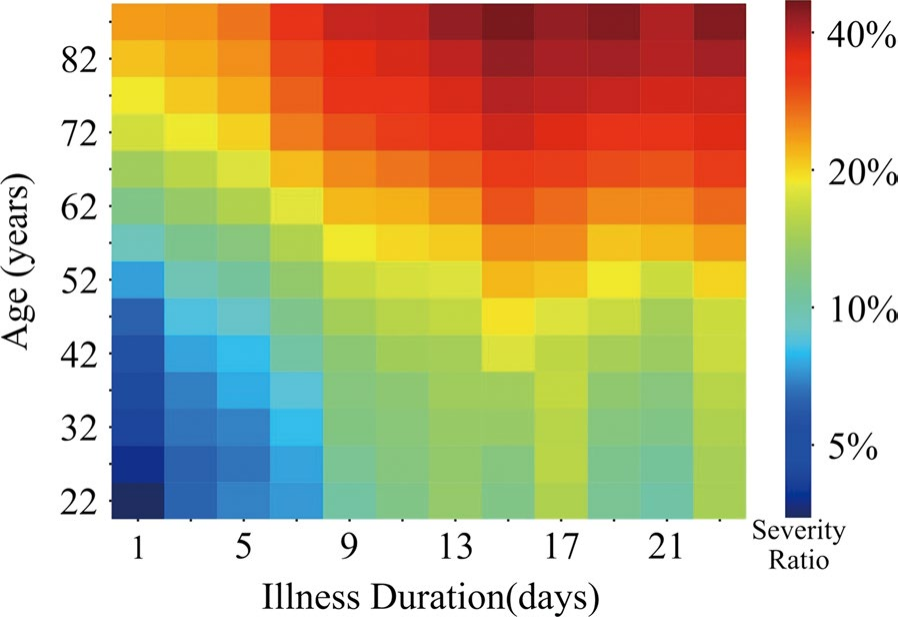
Distribution of Illness Severity by Age and Illness Duration. Severity Ratio refers to the proportion of patients with severe disease. This increased with advancing age and illness duration

Additional file 1: Figure S2 shows the distribution of disease severity by age and gender. Both age (r > 0·91, p < 0·0001) and illness duration (r > 0·69, p < 0·0001) correlated positively with disease severity, as seen in Fig. 1, and this was unaffected by gender (men and women held similar trend with age and illness duration as showed in Additional file 1: Figure S3). Patients with severe disease had a mean (SD) age of 60·85 (15·28) years and illness duration of 12·55 (7·93) days after symptom onset, compared with patients with nonsevere disease, who had a mean (SD) age of 51·90 (16·17) years and illness duration of 9·95 (7·82) days (t = 44·85, p < 0·0001 for age; t = 26·62, p < 0·0001 for illness duration). Patient location at time of enrollment did not differ significantly according to illness severity (χ^2^ = 0·17, p = 0·682) (Additional file 1: Table S1).

### Clinical symptoms and comorbidities

Clinical manifestations were recorded for 4,984 patients and yielded 20 clinical symptoms (Additional file 1: Table S2); among these, the incidence of dyspnea (χ^2^ = 24·56, p < 0·0001) and shortness of breath (defined as clinical evidence of altered breathing) (χ^2^ = 62·67, p < 0·0001) differed significantly between severe and nonsevere patients. Among 1,326 patients with severe disease, 225 (17·0%) had dyspnea and 296 (22·3%) had shortness of breath; conversely, among the 3,658 patients with nonsevere disease, 425 (11·6%) had dyspnea and 480 (13·1%) had shortness of breath.

Comorbid conditions were recorded for 5,062 patients and were found in a higher proportion of patients with severe versus nonsevere disease (Additional file 1: Table S3). Additionally, the number of comorbidities showed significant association with the severity of COVID-19 (t = 7·96, p < 0·0001). Patients with hypertension, pulmonary disease, diabetes mellitus, and cardio/cerebrovascular disease were more likely to develop severe disease (χ^2^ > 15·14, p < 0·0001). Notably, there was no significant difference in the prevalence of chronic liver (χ^2^ = 0·38, p = 0·538) or kidney disease (χ^2^ = 2·00, p = 0·157) between patients with severe and nonsevere disease.

### Laboratory and imaging results

Laboratory results were recorded for 2,471 patients. The percentages of neutrophils and lymphocytes were significantly different in patients with severe versus nonsevere disease: in patients with severe disease, neutrophils were higher (t =  − 7·53, p < 0·0001) and lymphocytes were lower (t = 4·67, p < 0·0001; Additional file 1: Table S4).

A total of 3,438 patients underwent computed tomography examination, revealing abnormalities in 90·5% of patients with severe disease and 92·2% of patients with nonsevere disease—a nonsignificant difference (χ^2^ = 2·16, p = 0·142).

### Predictor selection

Among the 36 variables analyzed, 12 showed statistical differences between the groups of patients with severe and nonsevere disease, indicating their potential predictive value: gender, age, illness duration, dyspnea, shortness of breath, hypertension, pulmonary disease, diabetes, cardio/cerebrovascular disease, number of comorbidities, neutrophil percentage, and lymphocyte percentage. Of these, four were strong predictors of severe disease (Table 1): age (odds ratio [OR] = 1·03; 95%CI: 1·02–1·04; p < 0·0001), illness duration (OR = 1·08; 95%CI: 1·06–1·10; p < 0·0001), shortness of breath (OR = 1·64; 95%CI: 1·26–2·13; p = 0·0002), and lymphocyte percentage (OR = 0·98; 95%CI: 0·97–0·99; p < 0·0001).

**Table 1:** Logistic Regression Model for the Prediction of Severe Disease

Variables	Odds Ratio	95% CI	t	*P* value
Age	1·03	1·02,1·04	7·28,	< 0·0001**
Gender	1·30	1·06,1·58	2·55	0·011
Illness Duration	1·08	1·06,1·10	9·12	< 0·0001**
Dyspnea	1·18	0·87,1·61	1·07	0·287
Shortness of Breath^†^	1·64	1·26,2·13	3·69	0·0002*
Hypertension	0·86	0·45,1·67	-0·43	0·664
Pulmonary Disease	1·67	0·73,3·81	1·23	0·220
Diabetes Mellitus	1·29	0·64,2·58	0·71	0·480
Cardio-cerebrovascular Disease	0·86	0·42,1·77	-0·41	0·684
No. of Comorbidities	1·03	0·57,1·85	0·10	0·921
L (Lymphocyte Percentage)	0·98	0·97,0·99	-4·79	< 0·0001**
N (Neutrophil Percentage)	1·00	0·99,1·00	-1·17	0·242

^†^clinical evidence of altered breathing

^*^: p < 0·01; **: p < 0·0001

### Effect size of strong predictors at different illness duration

**Fig. 2:**
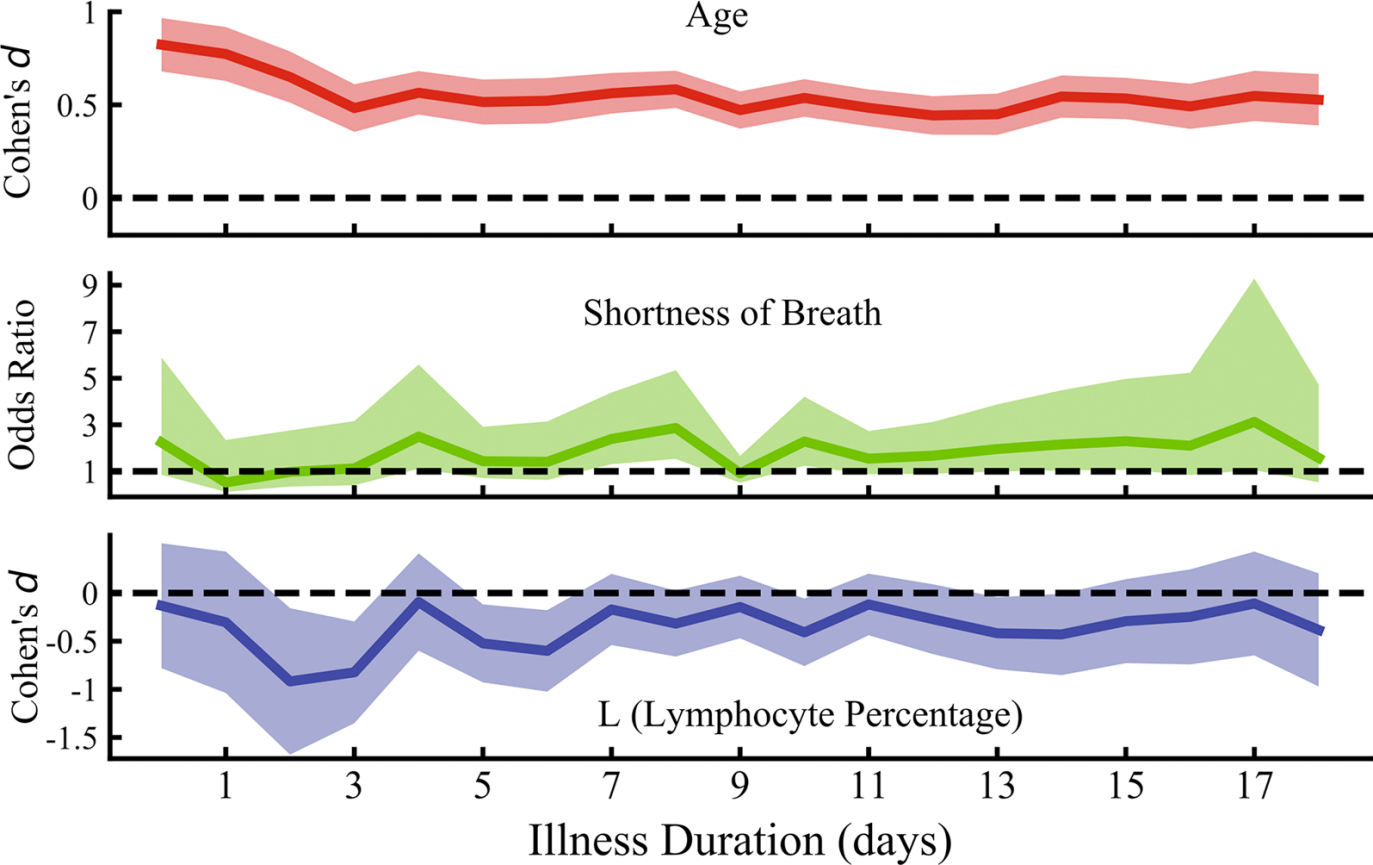
Effect size of strong predictors at different illness duration. The effect size (Cohen’s *d* for continuous variables and odds ratio for categorical variables) of three strong severity predictors (age, shortness of breath, and lymphocyte percentage) with different illness durations. All the three variables could help to find out severe patients at different illness duration

To further understand whether the above strong predictors have the same effect in different illness duration, we analyzed effect size of three strong severity predictors (age, shortness of breath, and lymphocyte percentage) at different illness duration. As sample size differed at different stages, we applied Cohen’s *d* for continuous variables and odds ratio for categorical variables as effect size to describe statistical results. As shown in Fig. 2, all the three variables were always helpful predictors to find out severe patients with illness duration increasing. The risk factors did not change with different illness duration.

### Construction and performance of the COVID-19 severity self-assessment scale

As described, regression modelling identified 12 variables for inclusion in the prediction scale; however, blood tests cannot be performed by patients doing self-assessment, and the two blood test indicators (neutrophil percentage and lymphocyte percentage) were excluded from the final scale, leaving a 10-item scale.

**Fig. 3:**
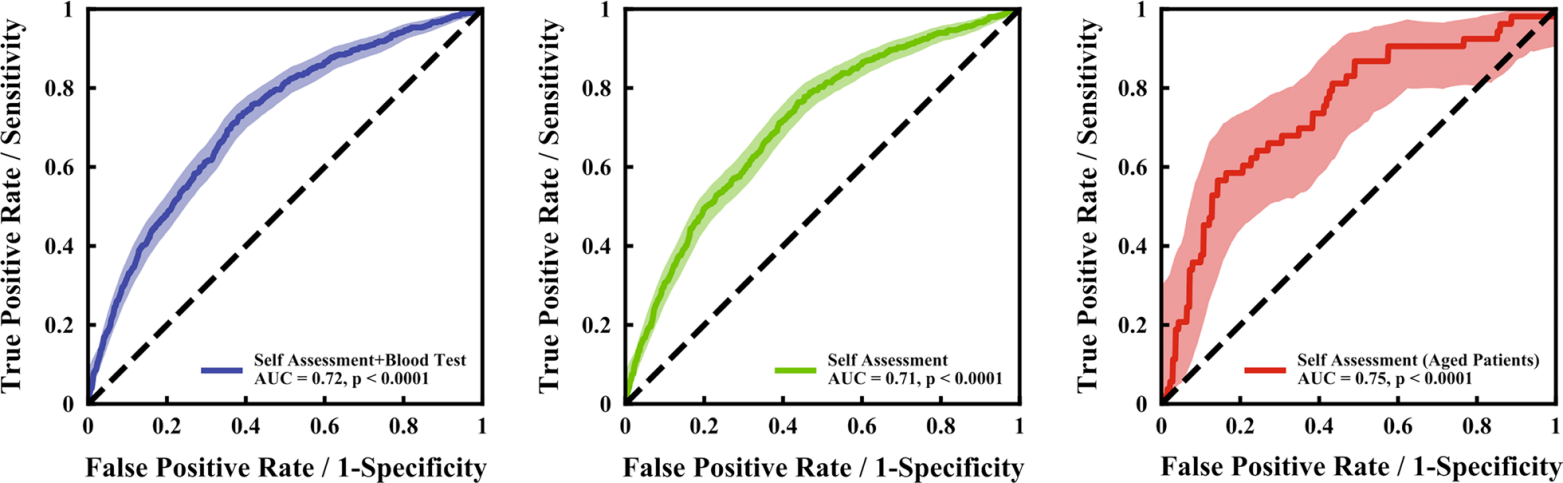
ROC Curves for Patients with Severe Disease. A ROC curve was generated for all 12 variables (i.e., 10 self-assessment variables and 2 blood test parameters) that showed strong correlation with disease severity (blue line, AUC = 0·72, p < 0·0001). Then, separate ROC curves were generated for the 10 variables in the final self-assessment scale (green line, AUC = 0·71, p < 0·0001) and for the same 10 variables, in the older-aged (not less than 65 years old) patients (red line, AUC = 0·75, p < 0·0001). ROC, receiver operating characteristic; AUC, area under the (ROC) curve

Figure 3 shows the results of the ROC analysis evaluating the accuracy of different model scales. The AUC of the full logistic regression model, with all 12 variables showing strong association to disease severity, was 0·72 (p < 0·0001). Following removal of neutrophil percentage and lymphocyte percentage, the remaining model (the final “COVID-19 Severity Self-Assessment Scale”) showed a similar AUC = 0·71 (p < 0·0001) and further, higher accuracy in older-aged (≥ 65 years) patients (AUC = 0·75, p < 0·0001). We also try to construct two-point scale, using just age and gender to predict the progression of COVID-19. The AUC was slightly lower (AUC = 0.65), but it’s easier to use.

The LASSO regression extracted similar results, indicating no confounding collinearity between the variables and cross-validating the prediction accuracy.

**Fig. 4:**
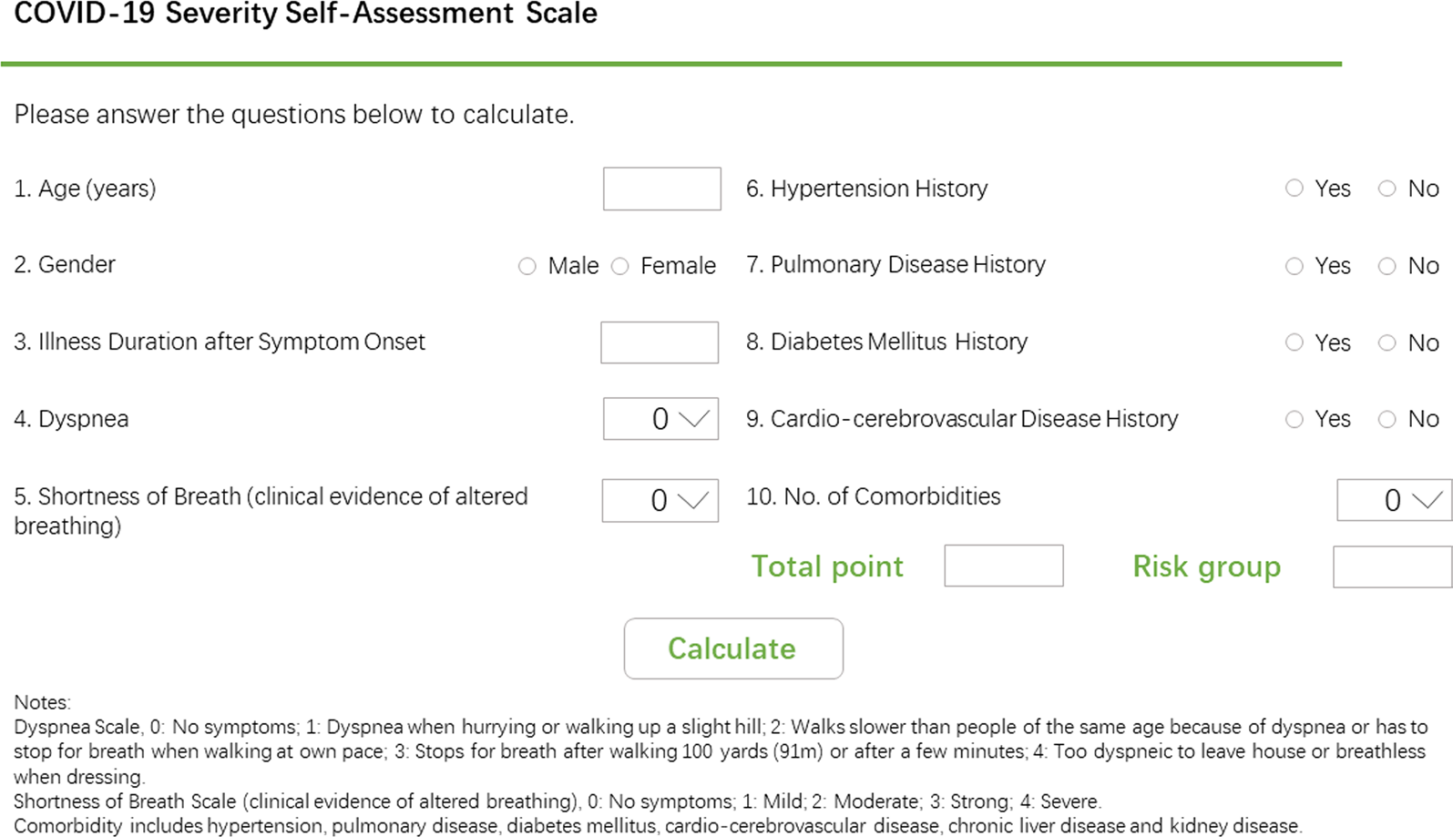
The Online Coronavirus Disease 2019 Severity Self-Assessment Scale. The scale is available at: http://180.167.250.222:10080/COVID-19-Severity-Self-Assessment-Scale.html. COVID-19, Coronavirus Disease 2019. Shortness of breath is defined here as clinical evidence of altered breathing

The final 10-item scale yielded a total score of 100 points, with higher score indicating a higher risk for severe illness. ROC analysis determined the cutoff value of 49·65, with scores above 49·65 predicting high risk. With this score, the final scale can correctly identify 87% patients. Once the predictive variables were determined and the self-assessment scale developed, an online calculator tool was constructed to allow patients access to expedient results (http://180.167.250.221:11080/COVID-19-Severity-Self-Assessment-Scale.html; Fig. 4).

## Discussion

The study results showed that age, illness duration after onset, shortness of breath, and lymphocyte percentage are key factors in predicting whether COVID-19 infection will advance to severe disease. Notably, patient location does not change the likelihood of developing a more serious illness. Fever is often thought to be associated with disease severity, such as in influenza, but we found no significant difference in fever rates between patients with severe and nonsevere COVID-19 disease. Previous studies [14, 21] have found comorbid chronic diseases to be correlated with increased disease severity among COVID-19 patients. A study outside Wuhan by Shi et al. also found that hypertension is a risk factor for severe COVID-19 [22]. Similarly, we found that four chronic diseases were more prevalent in patients with severe disease. While comorbidities showed weaker significance in the multivariate analysis, they were nevertheless included in the scale for higher accuracy of the model. Surprisingly, abnormality on chest computed tomography or X-ray exam did not distinguish patients with severe illness from those with nonsevere illness; however, our data only included the presence or absence of abnormality, and more detailed analysis of the abnormalities, e.g., of lesion size and distribution, might have disclosed different levels of severity—further analysis of the results by specialists is warranted [21, 23].

Previously, older age has been reported as an important risk factor in SARS and MERS [24, 25]. Our study confirmed that increased age was associated with COVID-19 severity and the severity risk would increase 1·32 times with every 10 years old. Older age has also been mentioned a strong relationship with death in patients with COVID-19 [26, 27]. Apart from age, we also found that illness duration after onset held a significant association with COVID-19 severity. The risk would increase 2·22 times with every 10 days after illness onset. Thus, we strongly recommend early detection and treatment in healthcare settings for patients with COVID-19, especially aged patients, to reduce the mortality rate. Shortness of breath, a symptom written in the Chinese Protocol on Prevention and Control of COVID-19 [19], was confirmed as a risk factor of COVID-19 severity in our study. Patients with shortness of breath held 1·64 times higher severity risk. Lymphocyte percentage, as a common blood test parameter, could be easily extracted in the community healthcare. There are several previous studies which mentioned that lymphocyte was a risk factor of COVID-19 severity [12, 28] with comparatively small sample sizes. In this study, we cross-validated this association with a large sample size and quantified that the severity risk would increase 1·26 times with 10% lymphocyte decreasing.

We developed a self-assessment tool to predict the development of severe illness among COVID-19 infected patients at home. To our knowledge, a list of risk scores has been established for COVID-19 mortality and severity. For example, a mortality risk prediction score was currently available online and methodologically suitable for use in the community [29]. However, it was not developed with COVID-19 patients’ data, and therefore further validation should be required. There are some studies that have developed similar critical illness risk score [30, 31], but they rarely pay attention to the effect of the illness duration on the severity of the disease. The possible reason is that the confirmed was immediately sent to a medical institution as soon as it was discovered, in the case of sufficient medical resources, such as in the late stage of the outbreak in China. We analyzed the data of the patients in the early stage of the outbreak in Wuhan and found that the longer they have been ill, the greater the possibility that the disease will develop into severe. Existing COVID-19 severity prediction models [26, 31–33] showed that complex laboratory indicators, such as direct bilirubin and lactate dehydrogenase, could help us to evaluate disease progression more accurately. On the other hand, results from these indicators relied a lot on the availability of specialized laboratory parameters. Furthermore, hospital visits would increase COVID-19 cross-infection risks. The COVID-19 Severity Self-Assessment Scale we constructed needn’t complex laboratory parameters and offers relatively high accuracy, which makes it convenient and practical for use in home self-assessment and for preliminary rapid screening in community healthcare settings.

To the best of our knowledge, this study included the most patients with COVID-19 before treatments in the early stage of the Wuhan outbreak. The lack of medical resources caused by the sudden outbreak prevented patients from receiving timely treatment, which resulted in a longer disease course (Additional file 1: Figure S1) that more closely resembled the natural progression of the disease. Despite these strengths, the data used in the scale construction were entirely from China, which could potentially limit the generalizability of the findings and use of the scale in other countries. Different sample size of each variable may weaken the final value of the scale, although the statistical model attempts to correct for this bias. The design of our study is not prospective, nor is there a longitudinal follow-up of a large cohort of outpatients with COVID-19, which deserve a further study. The possibility of presenting a severe COVID-19 may be related with the risk of death, but as we do not know the prognosis of the patients, our scale cannot be used to predict the risk of death.

## Conclusions

Twelve scale items—age, gender, illness duration, dyspnea, shortness of breath (clinical evidence of altered breathing), hypertension, pulmonary disease, diabetes, cardio/cerebrovascular disease, number of comorbidities, neutrophil percentage, and lymphocyte percentage—were identified and showed good predictive ability of whether confirmed patients would develop severe disease. After excluding the laboratory parameters, we constructed a COVID-19 severity self-assessment scale that can be used by patients in the community to predict their risk of developing severe illness and the need for further medical assistance. The tool is also practical for use in preliminary screening in community healthcare settings.

## Supplementary Information

**Additional file 1.** Supplementary Material.

## Data Availability

The datasets used and analyzed during the current study are not publicly available due to limitations of ethical approval involving the patient data and anonymity but are available from the corresponding author on reasonable request.
