# Supplementary material for: Progression of severity in coronavirus disease 2019 patients before treatment and a self-assessment scale to predict disease severity
Source: BMC Infect Dis. 2022 Apr 26;22:409. doi: 10.1186/s12879-022-07386-3 (PMC9040356; doi:10.1186/s12879-022-07386-3)
Supplement: Supplementary file 1 — Additional file 1. Supplementary Material. [file 12879_2022_7386_MOESM1_ESM.docx]

***Supplementary Material***

**Definitions of variables and outcomes**

Illness duration is defined as the number of days since the onset of symptoms when we found a patient. Quarantine status is a binary outcome. We defined the patient who was hospitalized or quarantined at cabin hospital or any centralized quarantine pot like hotel was quarantined, the patient at home was not quarantined.

Pulmonary disease refers to all chronic diseases related to the lungs, including diseases of the lungs themselves or pulmonary manifestations of systemic diseases. Diabetes mellitus refers to a variable disorder of carbohydrate metabolism caused by a combination of hereditary and environmental factors and usually characterized by inadequate secretion or utilization of insulin, by excessive urine production, by excessive amounts of sugar in the blood and urine, and by thirst, hunger, and loss of weight. Cardiovascular disease refers to several conditions including heart disease, heart attack, stroke, heart failure, arrhythmia, heart valve problems. Cerebrovascular disease is a heterogeneous disorder. It comprises of several distinct pathologies, including transient ischemic attack, stroke pathological types (ischemic stroke, intracerebral hemorrhage, subarachnoid hemorrhage) and etiological subtypes (e.g., cardioembolic, atherothrombotic, lacunar ischemic strokes, aneurysmal subarachnoid hemorrhage), and other intracranial vascular disorders (e.g., vascular malformations, unruptured aneurysms). Chronic liver disease is a set of diseases characterized by decreased hepatic function as a result of chronic inflammation or insult to the liver. Chronic kidney disease is defined as a progressive loss of renal function that lasts for more than 3 months, and is classified according to the degree of kidney damage – measured by the level of proteinuria – and the decline in glomerular filtration rate. The most severe form is end-stage renal disease.

The definitions of severe and critical COVID-19 pneumonia in this study are consistent with those in the Chinese Diagnosis and Treatment Protocol for COVID-19, which could be found at <http://www.nhc.gov.cn/xcs/zhengcwj/202003/46c9294a7dfe4cef80dc7f5912eb1989.shtml>.

The protocol defines four levels of COVID-19 disease: mild, ordinary, severe, and critical illness. The specific definitions are as follows:

Mild: The clinical symptoms were mild, and no pneumonia was found on imaging. Ordinary: Has symptoms such as fever and respiratory tract infection, and pneumonia can be seen on imaging. Severe: Adults has any of the following: a. Shortness of breath, RR≥30 times/min; b. In resting state, oxygen saturation≤93%; c. partial arterial pressure of oxygen (PaO2)/oxygen concentration (FiO2) ≤300mmHg (1mmHg=0.133kPa); In areas with high altitude (over 1000 meters above sea level), the PaO2/FiO2 should be corrected according to the following formula: PaO2/ FiO2×[atmospheric pressure (mmHg)/760].

Children has any of the following: a. Shortness of breath, (< 2 months old, RR≥60 times/min; 2–12 months old, RR≥50 times/min; 1-5 years old, RR≥40 times/min; >5 years old, RR≥30 times/min), excluding the effects of fever and crying; b. In resting state, oxygen saturation≤92%; c. Assisted breathing (groaning, alar flap, triple concave sign), cyanosis, intermittent apnea; d. Drowsiness, convulsions; e. Refusal to feed or feeding difficulties, with signs of dehydration. 4. critical illness: person with one of the following: a. Respiratory failure and need for mechanical ventilation; b. In shock; c. Combined with other organ failure, ICU monitoring and treatment are required.

**Table E1. Demographic Characteristics of the Sample**

|  | Severe,  mean (sd) / n (%)  (n=7798) | Nonsevere,  mean (sd) / n (%)  (n=37652) | t / χ^2^ | *P* value |
| --- | --- | --- | --- | --- |
| Age (years) | 60·85 (15·28) | 51·90 (16·17) | t = 44·85 | < 0·0001** |
| Gender (male) | 3908 (50·1%) | 17781 (47·2%) | χ^2^ = 21·64 | < 0·0001** |
| Illness Duration (days) | 12·55 (7·93) | 9·95 (7·82) | t = 26·62 | < 0·0001** |
| Quarantine (yes) | 6448 (82·7%) | 31206 (82·9%) | χ^2^ = 0·17 | 0·682 |

Abbreviation: sd, standard deviation

**: p<0·0001.

**Table E2. Reported Symptoms of the Participants**

| Symptoms | Severe, n (%) (n=1326) | Nonsevere, n (%) (n=3658) | χ^2^ | *P* value |
| --- | --- | --- | --- | --- |
| Fever | 1110 (83·7) | 2951 (80·7) | 5·95 | 0·015 |
| Vomiting | 72 (5·4) | 160 (4·4) | 2·44 | 0·118 |
| Dyspnea | 225 (17·0) | 425 (11·6) | 24·56 | < 0·0001** |
| Shortness of Breath^†^ | 296 (22·3) | 480 (13·1) | 62·67 | < 0·0001** |
| Expectoration | 289 (21·8) | 715 (19·5) | 3·06 | 0·080 |
| Sore Throat | 55 (4·1) | 229 (6·3) | 8·08 | 0·004* |
| Headache | 129 (9·7) | 463 (12·7) | 7·97 | 0·005* |
| Chills | 126 (9·5) | 372 (10·2) | 0·48 | 0·488 |
| Dry Cough | 580 (43·7) | 1661 (45·4) | 1·09 | 0·296 |
| Nausea | 36 (2·7) | 100 (2·7) | 0·00 | 0·971 |
| Runny Nose | 19 (1·4) | 101 (2·8) | 7·31 | 0·007* |
| Conjunctival Hyperemia | 3 (0·2) | 7 (0·2) | 0·06 | 0·808 |
| Muscle Soreness | 237 (17·9) | 658 (18·0) | 0·01 | 0·926 |
| Chest Pain | 22 (1·7) | 99 (2·7) | 4·51 | 0·034 |
| Chest Tightness | 229 (17·3) | 551 (15·1) | 3·59 | 0·058 |
| Diarrhea | 127 (9·6) | 362 (9·9) | 0·11 | 0·738 |
| Abdominal Pain | 3 (0·2) | 14 (0·4) | 0·70 | 0·402 |
| Nasal Congestion | 23 (1·7) | 74 (2·0) | 0·42 | 0·515 |
| Fatigue | 537 (40·5) | 1335 (36·5) | 6·65 | 0·010* |
| Joint Soreness | 60 (4·5) | 224 (6·1) | 4·63 | 0·031 |

^†^clinical evidence of altered breathing

*: p<0·01; **: p<0·0001.

**Table E3. Reported Comorbidities of the Participants**

| Comorbidities | Severe,  n (%) / mean (sd)  (n=1339) | Nonsevere,  n (%) / mean(sd)  (n=3723) | t / χ^2^ | *P* value |
| --- | --- | --- | --- | --- |
| Hypertension | 306 (22·9%) | 599 (16·1%) | χ^2^ = 30·69 | < 0·0001** |
| Pulmonary Disease | 51 (3·8%) | 71 (1·9%) | χ^2^ = 15·14 | < 0·0001** |
| Diabetes Mellitus | 147 (11·0%) | 239 (6·4%) | χ^2^ = 29·06 | < 0·0001** |
| Cardio-cerebrovascular Disease | 126 (9·4%) | 214 (5·7%) | χ^2^ = 21·08 | < 0·0001** |
| Chronic Liver Disease | 14 (1·0%) | 32 (0·9%) | χ^2^ = 0·38 | 0·538 |
| Chronic Kidney Disease | 17 (1·3%) | 31 (0·8%) | χ^2^ = 2·00 | 0·157 |
| No. of Comorbidities^†^ | 0·50 (0·80) | 0·32 (0·66) | t = 7·96 | < 0·0001** |

^†^total number of comorbid conditions, including hypertension, pulmonary disease, diabetes mellitus, cardio/cerebrovascular disease, chronic liver disease, and chronic kidney disease, for each subject

**: p<0·0001.

**Table E4. Reported Laboratory Results of the Participants**

| Indexes | Severe, mean(sd) (n=605) | Nonsevere, mean(sd) (n=1866) | t | *P* value |
| --- | --- | --- | --- | --- |
| WBC (White Blood Cell Count) | 5·69 (3·07) | 5·37 (2·87) | 2·38 | 0·018 |
| Lx (Lymphocyte Count) | 1·33 (3·47) | 1·96 (6·19) | -2·39 | 0·017 |
| L (Lymphocyte Percentage, %) | 20·21 (12·74) | 24·90 (13·50) | -7·53 | < 0·0001** |
| N (Neutrophil Percentage, %) | 67·17 (21·24) | 62·90 (18·94) | 4·67 | < 0·0001** |

**: p<0·0001.


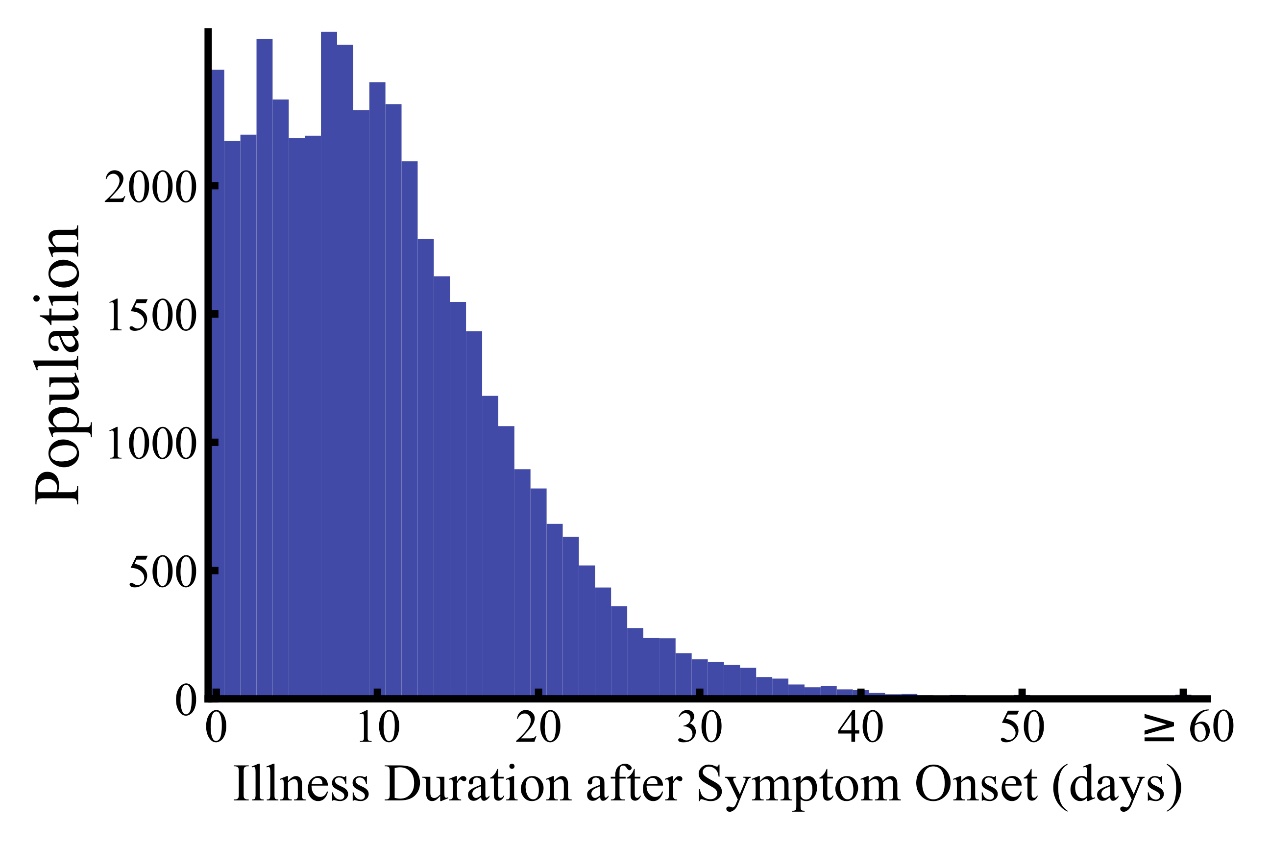


**Figure E1. Illness Duration in the Study Population.**

This picture shows the distribution of the duration of the disease after the onset of symptoms in the studied patients.


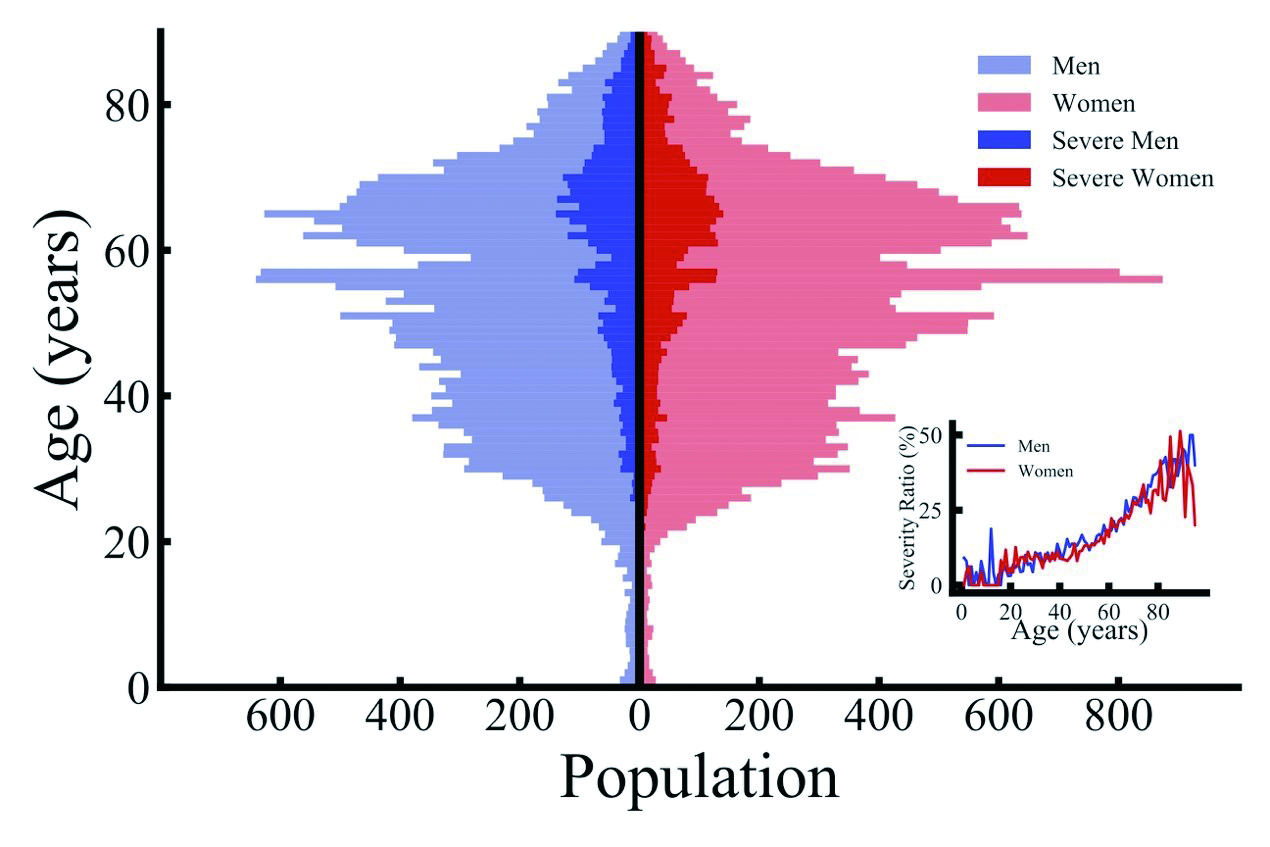


**Figure E2. Distribution of Disease Severity by Age and Gender.**

Severity Ratio refers to the proportion of patients with severe disease. Age correlated positively with Coronavirus Disease 2019 severity (r>0·91, p<0·0001), and this was unaffected by gender.

Severity Ratio refers to the proportion of patients with severe disease.


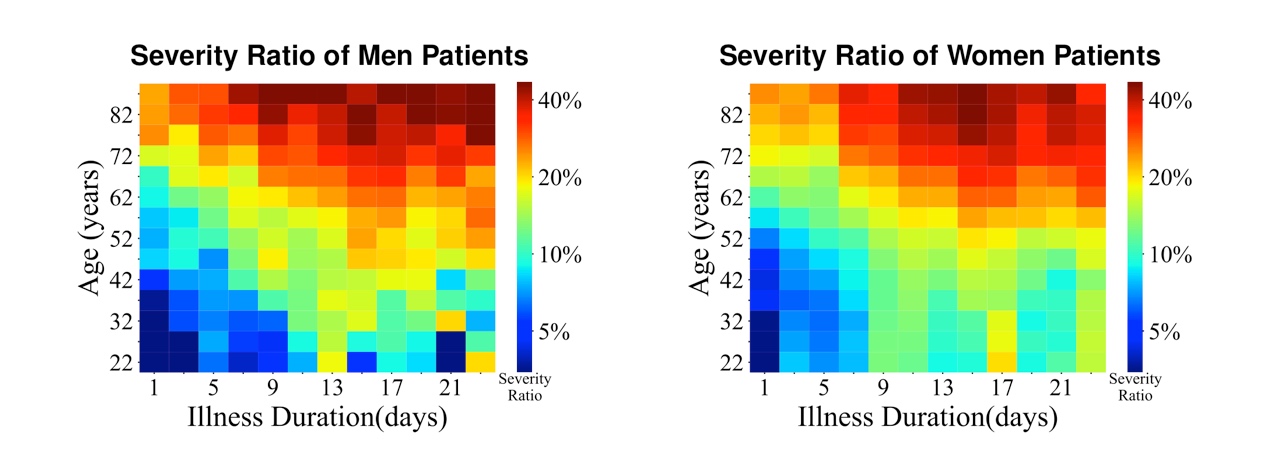


**Figure E3. Severity Ratio at Different Ages and Illness Durations, in Men and Women.**

Severity Ratio refers to the proportion of patients with severe disease.
